# On the waiting list for joint replacement for knee osteoarthritis: Are first-line treatment recommendations implemented?

**DOI:** 10.1016/j.ocarto.2020.100056

**Published:** 2020-03-10

**Authors:** A. Cronström, H. Nero, L.S. Lohmander, L.E. Dahlberg

**Affiliations:** a Orthopaedics, Department of Clinical Sciences Lund, Lund University, Lund, Sweden; b Department of Health Sciences, Lund University, Lund, Sweden; c Department of Community Medicine and Rehabilitation, Umeå University, Umeå, Sweden

**Keywords:** Osteoarthritis, Joint replacement, First-line treatment, Implementation, Exercise, Education

## Abstract

**Objective:**

To investigate to what extent individuals participated in guideline-based first-line treatments before being assigned to a wait list for knee replacement for osteoarthritis (OA), and to what extent they were recommended such treatments once on the list. Factors associated with participation in first-line management were also investigated.

**Design:**

All patients on the waiting list ≥ three months for knee replacement due to knee OA (n = 229) at a public hospital in Sweden were invited to participate in this cross-sectional survey study. 136 individuals (mean age 70 ± 9 years, 59% women) answered self-reported questionnaires including demographics, physical activity level, knee function and treatments before and during their time on the waiting list.

**Results:**

Before being referred to the waiting list, 40% had participated in guideline-based OA management (Better management of patients with OsteoArthritis (BOA)), 53% in physiotherapy, 67% in either BOA or physiotherapy whilst 23% of those overweight (BMI≥25) had received weight-management advice. Women had participated in BOA and physiotherapy twice as often as men (51% vs. 25%, p = 0.002 and 66% vs. 34%, p < 0.001) prior to waiting list referral. During their time on the waiting list, only 10% were recommended BOA, 30% physiotherapy and 15% weight-management. 38% of the patients that had never participated in BOA indicated that they were interested in participating while waiting for their knee replacement.

**Conclusion:**

Our results suggest that recommended treatment guidelines for OA may not be adequately implemented in Swedish health-care. Further exploration of implementation barriers and lack of equality of care appears warranted.

## 1 Introduction

Osteoarthritis (OA) is one of the fastest growing causes of disability worldwide with a prevalence of 10–15% [[1], [2], [3]]. In the US, more than 30 million individuals suffer from OA at an annual cost of 100 billion US dollars yearly, of which knee OA alone stands for 27 billion US dollars, largely due to the high use of knee replacement surgery [4]. While the incidence of knee replacement surgery differs between countries [5], studies in Spain and the US have reported approximately one third of the performed joint replacements to not be clinically indicated, i.e., the patients showed only mild to moderate OA symptoms when the surgery was performed [6,7]. Moreover, approximately one in five patients is not satisfied with the outcomes after knee replacement surgery [8,9]. The increasing OA prevalence and difficulties to select patients, due to differences in surgery indication criteria among physicians [10,11] and that willingness for surgery may be the major indicator for surgery [12,13] provides a rationale for improved patient evaluation and optimization.

First-line OA management, including education, exercise and weight-control if indicated, has been shown to improve pain, physical function and quality of life both short- and long-term in patients with hip and knee OA [[14], [15], [16], [17],^19^], as well as to reduce or delay the need for joint replacements [[20], [21], [22], [23], [24], [25]]. Such treatment is commonly delivered through coordinated programs such as the *Better management of patients with OsteoArthritis* (BOA) [[14], [15], [16]] and *Good Life with osteoArthritis* in Denmark (GLA:D®) [17,18]. BOA is an OA management concept, including education and an option to physiotherapist-led or home-based exercise, offered to patients with confirmed clinical and/or radiological OA at some 600 primary care clinics across Sweden since 2008. Patients can be included by referral or self-referral. The BOA concept is part of the Swedish exemption card meaning that cost is deductible after an annual co-payment of 1150 SEK (around 100 Euro). Exercise while on the waiting list for joint replacement is reported to be safe, is well tolerated and reduces pain and improves function pre-operatively as well as post-operatively [[26], [27], [28]]. Receiving first-line treatment such as GLA:D® while on the waiting list for joint replacement may also delay the need for surgery [21]. Yet, international studies suggest that guidelines for such first-line treatment may not be well implemented [29,30]. We do not know to what extent recommended first-line management, as exemplified by the BOA concept, is implemented in Swedish OA care, or to what extent these treatment options are recommended to the patients while waiting for their knee replacement surgery. The aims of this study were to investigate participation in first-line OA management prior to referral to a waiting list for knee joint replacement, and, to what extent such management was recommended once on the list, of individuals on a knee replacement waiting list for 3 months or longer at a Swedish Hospital. We also explored associations between demographic factors, physical activity level, knee function and quality of life, and participation in first-line treatments prior to surgery referral as well as being recommended such treatment while on the waiting list.

## 2 Methods

This study is a cross-sectional survey study and was conducted according to the STROBE guidelines for observational studies [31]. In Sweden there is a nationally implemented Health Care Guarantee of a maximum of 90 days between patients’ first visit and the day of surgery. This is, however, not always realized in the real-world health care and patients often wait significantly longer for their surgery [32]. To be able to answer the question of what treatments the patients are offered during this waiting, we only included participants that had been on the waiting list ≥3 months.

### 2.1 Participants

**Fig. 1:**
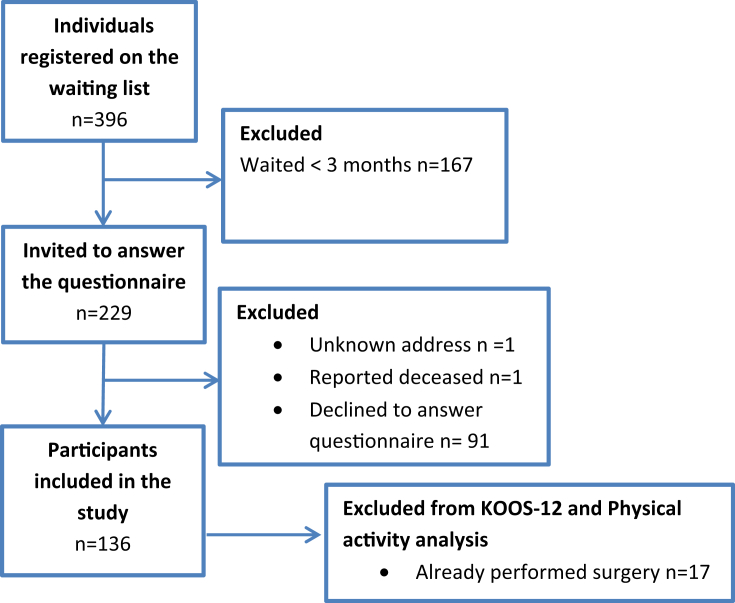
Flowchart of the inclusion process.

On the 19^th^ of February 2019, 396 individuals were registered on the waiting list for knee joint replacement due to knee OA at a public hospital in southern Sweden. Of these, 229 (58%) had been on the waiting list for 3 months or more and were invited by regular mail to answer questionnaires regarding demographics, function and other treatments. The exclusion criterion was being not able to understand the Swedish language. One invitation was returned due to unknown address and one participant was reported deceased. Of the remaining 227 individuals, 136 (60%) individuals gave their written informed consent to participate and answered the questionnaire (see Fig. 1 for a flow chart of the inclusion process). The study was approved by The Regional Ethical Review Board in Lund, Sweden (Dnr 2018/939).

### 2.2 Data collection

Age, sex and current time on the waiting list were retrieved from the hospital patient register. The following data were collected through a patient questionnaire: i) anthropomorphic and sociodemographic data including weight, height, working status, educational level and comorbidities; ii) measures of physical activity and knee function; iii) questions regarding participation in first-line OA treatments before the decision about surgery (e.g. BOA, physiotherapy, weight-management, walking aids, analgesics); iv) first-line OA treatments recommended whilst waiting for surgery and v) if they were interested in participating in BOA, if they had not previously participated in such an OA management program. The question about physiotherapy was “have you participated in/or been recommended physiotherapy prior to or during your time on the waiting list?” and consequently, physiotherapy was not specifically defined. All questions on treatments utilized prior to surgery referral and treatments being recommended once on the waiting list were asked with regards to management of their OA symptoms (Appendix A).

Knee function and knee-related quality of life were evaluated using the Knee injury and Osteoarthritis Outcome Score (KOOS-12) [33,34]. The KOOS-12 is a questionnaire of 12 items measuring knee pain, physical function and knee related quality of life, a short version of the original KOOS [35], and intended to capture the person's opinion about the experienced difficulties of activity due to knee problems. All items were scored from 0 to 4. The scores were then normalized to a total score from 0 to 100, where 0 indicates extreme problems and 100 indicates no problems. Level of physical activity was assessed using the Swedish Board of Health and Welfare indicator questions, which consist of two questions; patient-defined minutes of physical activity and minutes of exercise, both defined as per week [36]. The minutes reported were then converted to a score from 1 to 7 and 1–6, respectively. A total score of physical activity is then calculated by taking the physical activity score∗2 + the exercise score, leaving a total score range of 3–19, where a score <11 indicates a physical activity level below the recommended 150 min per week [36].

### 2.3 Statistics

We performed statistical analysis using SPSS version 25 (IBM Corporation, New York, USA). Data were checked for normality using visual inspection of histograms and interpretation of skewness and kurtosis. All data met the assumptions of normality except number of days spent on the waiting list. The independent t–test was used to evaluate difference in age, the Mann-Whitney *U* test for difference in waiting time and cross-tabulation and chi^2^ for sex differences between those who responded to the questionnaires and those who did not. Cross-tabulation and chi^2^ (categorical data) and the independent *t*-test (continuous data) were used to evaluate possible associations between the participants’ characteristics and participation in first-line treatment before being referred to the waiting list for joint replacement and first-line treatment recommended while on the waiting list. Cross-tabulation, chi^2^ and the independent *t*-test were also used as appropriate to evaluate any associations between participant characteristics and the interest in participating in BOA while on the waiting list. A p-value less or equal to 0.05 was considered statistically significant.

## 3 Results

There were no statistically significant differences in the number of days on the waiting list (Median (q1-q3)) (132 (111–167) vs (127 (111–166), p = 0.597), mean (SD) age (70 (8.6) vs 71 (10), p = 0.210) or distribution of sex (59% vs 49% women, p = 0.140) between the individuals that answered the questionnaires and those who did not. (See Table 1 for all characteristics of the included participants). Seventeen participants (12.5%) had already undergone surgery when receiving the questionnaire and these individuals were not included in the analyses for KOOS-12 and physical activity level.

**Table 1:** Characteristics of the included participants (n = 136).

Characteristic	
Sex (women) n (%)	80 (58.8)
Age mean (sd)	70 (8.6)
BMI mean (sd)	30.1 (6.6)
Working status	
Working n (%)	22 (16.2)
Retired n (%)	102 (75.0)
Sick-leave n (%)	10 (7.4)
Unemployed n (%)	2 (1.5)
Education level	
None	1 (0.7)
Elementary school n (%)	38 (27.9)
High school n (%)	51 (37.5)
College/University n (%)	46 (33.8)
Comorbidity n (%)	85 (62.5)
Heart disease n (%)	36 (26.5)
Lung disease n (%)	18 (13.2)
Diabetes n (%)	26 (19.1)
Chronic pain/fibromyalgia n (%)	17 (12.5)
Other health condition n (%)	39 (28.7)
KOOS-12 mean (sd) (n = 119)	29.2 (15.1)
Physical activity level mean (sd) (n = 119)	9.7 (4.8)
Current time on waiting list in days median (quartiles)	132 (111–167)
Type of Surgery	
Total knee replacement	128 (93.6)
Unicompartmental knee replacement	8 (5.8)
Performed surgery n (%)	17 (12.5)

BMI = body mass index, physical activity level: a score < 11 indicates a physical activity level below the recommended 150 min per week.

### 3.1 First-line treatment prior to knee joint replacement referral

Before being referred to the waiting list for surgery, the participants reported to have utilized the following first-line treatments: BOA program n = 55 (40%), physiotherapy n = 72 (53%), walking aid n = 51 (38%) and analgesics n = 71 (52%). Of the participants that had a BMI of ≥25 (n = 102), 23 participants (23%) reported to have received weight-management advice. Ninety-one participants (67%) participated in either the BOA program or physiotherapy and 81 (60%) participants had used at least two of the different management options prior to surgery referral.

A higher proportion of women had participated in BOA (51% vs 25%) or physiotherapy (66% vs 34%) before being referred to surgery (Table 2). The participants that had participated in BOA had a lower (worse) KOOS-12 score than those who had not participated in BOA (25 (13.7) vs 32 (15.4)) and those who had participated in physiotherapy before surgery referral had lower KOOS-12 score than those who had not (26 (13.3) vs 32 (16.3)) (Table 2). No other important differences in participants’ characteristics were found between those who had participated in BOA and/or physiotherapy prior to knee joint replacement referral and those who had not (Table 2).

**Table 2:** Differences in patients’ characteristics between those who participated in BOA or physiotherapy before being referred to knee replacement and those who did not and between those who were recommended physiotherapy while waiting and those who were not.

Characteristic	Participated in BOA	P-value	Participated in physiotherapy	P-value	Recommended physiotherapy during waiting	P-value
n = 136						
Sex						
Women %	51.3	**0.002**^a^	66.7	**<0.001**^a^	37.5	**0.026**^a^
Men %	25.0		33.9		19.6	
Age (years) mean (sd)	Yes: 70 (8.9)	0.954^b^	Yes: 69 (8.9)	0.094^b^	Yes: 69 (7.9)	0.361^b^
	No: 70 (8.1)		No: 71 (8.0)		No: 70 (8.9)	
BMI	Yes: 30.3 (6.0)	0.779	Yes: 29.6 (5.2)	0.413^b^	Yes: 31.0 (8.3)	0.296^b^
	No: 30.0 (7.1)		No: 30.6 (8.0)		No: 29.7 (5.7)	
Working status %						
Working	27.3	0.199^a^	54.5	0.323^a^	31.8	0.779^a^
Retired	42.2		70		29.4	
Sick-leave	60		52		40.0	
Education level %						
Elementary school	31.6	0.466^a^	50	0.123^a^	31.6	0.067^a^
High school	45.1		43.1		19.6	
College/University	43.5		65.2		41.3	
Comorbidity %						
Yes	42.4	0.558^a^	54.1	0.723^a^	30.6	0.885^a^
No	37.3		51		29.4	
Heart disease %						
Yes	41.7	0.861^a^	44.4	0.234^a^	22.2	0.227^a^
No	40.0		56		33.0	
Lung disease %						
Yes	44.4	0.710^a^	50	0.788^a^	22.2	0.432^a^
No	39.8		53.4		31.4	
Diabetes %						
Yes	42.3	0.829^a^	61.5	0.329^a^	30.8	0.939^a^
No	40.0		50.9		30.0	
Chronic pain/fibromyalgia %						
Yes	47.1	0.552^a^	64.7	0.299^a^	23.5	0.525^a^
No	39.5		51.7		31.1	
Other health condition %						
Yes	46.2	0.389^a^	53.8	0.893^a^	35.9	0.354^a^
No	38.1		52.6		27.8	
n = 119						
KOOS-12 mean (sd)	Yes: 25.0 (13.7)	**0.013**^b^	Yes: 26.2 (13.3)	**0.020**^b^	Yes: 29.9 (15.0)	0.747^b^
	No:31.9 (15.4)		No: 32.7 (16.3)		No: 28.9 (15.2)	
Physical activity level mean (sd)	Yes: 9.2 (4.7)	0.363^b^	Yes: 9.9 (5.0)	0.517^b^	Yes: 10.9 (4.1)	0.052^b^
	No: 10.0 (5.1)		No: 9.4 (4.7)		No: 9.1 (5.1)	

BOA=Better management of patients with osteoarthritis, BMI = body mass index, KOOS=Knee osteoarthrtis Outcome Score, physical activity level: a score < 11 indicates a physical activity level below the recommended 150 min per week.

Bold characters indicate significance.

a Chi-square test.

b Independent *t*-test.

### 3.2 First-line treatments while on the waiting list

Only 8 participants (10%) of those who had not participated in BOA (n = 81) prior to surgery referral were recommended BOA while on the waiting list for surgery. Forty-one (30%) of the 136 participants were recommended physiotherapy, n = 24 (18%) walking aids, n = 34 (25%) analgesics and n = 15 (15%) of the 102 over-weight (BMI≥25) patients were recommended weight-management while on the waiting list.

Due to the small number of participants (n = 8) that were recommended BOA once on the list, this variable was excluded from further analysis on associated factors for being recommended such treatment while on the waiting list. A higher proportion of women (38% vs 20%) had been recommended physiotherapy while waiting for joint replacement. No other differences between those who had been recommended physiotherapy once on the list and those who had not were observed (Table 2).

### 3.3 Interest in participating in BOA while waiting for surgery

24 of the 64 participants (38%) that did not participate in BOA before surgery referral and were not recommended to participate during their time on the waiting list answered that they would like to participate in the BOA program while waiting for surgery. There were no relevant associations between any of the participant characteristics and the interest in participating in BOA while on the waiting list (Table 3).

**Table 3:** Differences in patients’ characteristics between those who wished to participate in BOA while waiting for knee replacement and those who did not.

Characteristic	Wish to participate in BOA	p-value
n = 64		
Sex %		
Women	37.9	0.980^a^
Men	38.2	
Age (years) mean (sd)	Yes = 71.4 (10.4)	0.656^b^
	No = 70.3 (8.4)	
BMI mean (sd)	Yes: 29.9 (6.8)	0.682^b^
	No: 30.7 (7.9)	
Working status %		
Working	58.3	0.158^a^
Retired	37.0	
Sick-leave	0	
Education level %		
Elementary school	40	0.886^a^
High school	40	
College/University	33.3	
Comorbidity %		
Yes	40.0	0.681
No	34.8	
Heart disease %		
Yes	47.1	0.373^a^
No	34.8	
Lung disease %		
Yes	44.4	0.672^a^
No	37.0	
Diabetes %		
Yes	50	0.345^a^
No	35.3	
Chronic pain/fibromyalgia %		
Yes	37.5	0.970^a^
No	38.2	
Other health condition %		
Yes	22.2	0.101^a^
No	44.4	
n = 57		
KOOS-12 mean (sd)	Yes: 30.4 (16.0)	0.523^b^
	No: 32.9 (13.2)	
Physical activity level mean (sd)	Yes: 10.0 (4.8)	0.698^b^
	No: 9.4 (5.1)	

BOA=Better management of patients with osteoarthritis, BMI = body mass index, KOOS=Knee osteoarthritis Outcome Score, physical activity level: a score < 11 indicates a physical activity level below the recommended 150 min per week.

Bold characters indicate significance.

a Chi-square test.

b Independent *t*-test.

## 4 Discussion

In the presence of consistent guidelines for first-line OA management [[37], [38], [39]], only 2 out of 5 participants in the current study had participated in the BOA program, and 2 out of 3 in either BOA or physiotherapy, before being referred to surgery. Few were recommended first-line treatment while on the waiting list. Women had participated in the BOA program or physiotherapy twice as often as men before being assigned to the waiting list for surgery. If the results from this study are representative for other Swedish health-care regions, the recommended guidelines for first-line OA treatment are not adequately implemented in Swedish health-care.

Our results from this Swedish cohort are comparable to a recent systematic review, including studies from the US, Europe and Australia, where only about one in three OA patients were offered exercise and education [29], and Canada where 60% had undergone first-line treatment prior to surgery referral [40]. A recent study from Denmark reported that 37% of patients with OA had consulted a physical therapist prior to consulting an orthopaedic surgeon [30]. To our knowledge, ours is the first study to investigate what management options are recommended to the patients while on the waiting list for knee replacement. Although pre-surgery exercise may reduce the need for surgery [21] and is known to improve post-surgery function and reduce hospitalization time after joint replacement [41], only 10% and 30% of the patients in the present study were recommended BOA and physiotherapy, respectively, while on the waiting list for knee replacement. Specifically, exercising would be beneficial for patients that already have a low physical activity level [42]. Our results indicate that there is room for improvement of implementation of current guidelines both prior to surgery referral and once on the waiting list, specifically at those hospitals where prolonged time is spent waiting for surgery.

Women were twice as likely to have participated in BOA treatment and physiotherapy than men, whereas there were no differences in other demographics (age, BMI, comorbidities, working status, education, physical activity level) between those who had participated in BOA or physiotherapy and those who had not. This is in line with a recent study from Canada where men participated in first-line management to a lesser extent than women prior to knee replacement referral [40]. We speculate that women may have been referred to such treatment more often, or that men more often declined this option. In support of the first assumption, previous research concluded that despite being equally willing to have their joints replaced and having the same OA severity, women are less likely to be referred to surgery compared to men [12]. In the current study, women were more likely to be recommended physiotherapy once on the list compared to men, but men and women were equally willing to participate in the BOA concept while waiting for surgery. Further studies on possible reasons for the disparity in the recommendation and participation in first-line treatments between men and women are warranted.

Of further interest in this context is that worse KOOS-12 scores were associated with having participated in BOA and/or physiotherapy prior to surgery referral, and that women had significantly worse KOOS-12 score than men (27 vs 33, p = 0.027). That women participated in BOA/physiotherapy to a greater extent than men and are reported to have lower KOOS score than men [43] may be one explanation for the association between worse KOOS-12 score and participation in first-line treatment. Another explanation may be related to change in willingness for knee replacement after participation in first-line treatment. In our previous studies, participants changed their attitude to surgery, in both directions, after completion of a self-management program for OA, depending on the success of the program to reduce the OA symptoms [24,25]. Perhaps the participants in the current study that improved their OA symptoms with the management program were not referred to surgery or were no longer interested in surgery, while those who did not improve or got worse were put on the knee replacement waiting list. This could explain the association between worse KOOS-score and previous participation in BOA or physiotherapy, and would be consistent with the findings of the MEDIC study [21] where participation in pre-surgery exercise delayed the need for knee replacement in approximately 70% of the knee OA patients.

Weight management is an important part of first-line OA management. In the present study only 22% of the over-weight patients received weight-management advice prior to surgery referral, and only 15% were recommended such management once on the waiting list. This is in line with previous studies where 11–30% of over-weight patients with OA were reported to have received weight management and dietary treatment [44,45]. A combination of exercise and weight loss has been shown to decrease the level of inflammation, reduce compressive knee force and pain as well as improve self-efficacy and physical function compared to exercise alone in patients with OA [46,47]. Hence, if indicated, weight management should be considered as an important part of the OA treatment in this group of patients. If the effect of exercise and weight loss is not sufficient, walking aids and analgesics should be added to the treatment [48]. In the current study, only 37% and 52% received walking aids and analgesics prescriptions, respectively, prior to surgery referral, further highlighting the need for improvement in the recommendations of first-line treatment options.

Previous studies have sought to identify patients' barriers to first-line treatment [[49], [50], [51], [52]] as well as barriers to the clinical practitioners’ recommendations of first-line OA treatment [53,54]. Barriers related to accessibility of OA treatment at primary care clinics [[49], [50], [51]] and a belief that knee joint loading and exercise would cause further impairment and pain [52] is commonly reported among patients with OA. The most common reasons for not recommending these options among health-care professionals were reported to be lack of knowledge of current treatment guidelines as well as a belief that physiotherapy and exercise are not effective for OA [53,54]. Thus, future studies should focus on how to overcome these barriers for both patients and health-care professionals.

This study has several limitations. The response rate was only 60%. In part, this may be explained by a few weeks passing between the data extraction from the surgery register and distribution of questionnaires and that we do not know how many declined to answer the questionnaire because they had already had their surgery. Of those that responded, 17 individuals indicated that they had had surgery and were not included in the result for KOOS-12 and physical activity level. There was no difference in sex, age and time spent on the waiting list between those who agreed to participate and those who did not respond. “Physiotherapy” was not specifically defined in the questionnaire with regard to type, intensity, frequency or number of sessions. Different participants may have interpreted physiotherapy differently and it is possible that they have received acupuncture or other passive treatments instead of first-line treatment. This study included participants from one hospital in southern Sweden. However, the BOA concept is offered at primary care clinics across Sweden and the participants in this study reflect the general Swedish population on waiting lists for knee replacement with regard to sex, age and BMI and may thus be a representative sample [55]. We therefore suggest that the result from this study is generalizable across Swedish health-care regions. Results are also in agreement with findings internationally [29]. All data and information in this study (except sex, age and time on waiting list) were self-reported. Hence, some information, especially regarding what other treatments had been recommended, may be influenced by recollection bias. Finally, since this was an exploratory study, we did not adjust for multiple comparisons [56]. Given the number of statistical tests performed, it is possible that the associations between sex and KOOS-12 score and the participation and/or recommendation of first-line treatment is the result of chance.

The results from this study suggest that current OA treatment guidelines may not be adequately implemented. Given the observed interest among patients to participate in first-line treatment while waiting for surgery, future studies should focus on how to overcome barriers to recommend such OA treatment both prior to surgery referral and while on the waiting list for knee replacement. The apparent sex differences in participation and received recommendations for first-line treatments need attention to ensure equality in health care for men and women suffering from knee OA.

## Authors’ contribution

Anna Cronström contributed to the conception and design of the study, was responsible for the acquisition, analysis and interpretation of the data and was in charge of writing the manuscript. Håkan Nero contributed to the conception and design of the study, contributed to the analysis and interpretation of the data, and contributed to the drafts of this paper. L Stefan Lohmander contributed to the conception and design of the study, contributed to the analysis and interpretation of the data, and contributed to the drafts of this paper. Leif E Dahlberg contributed to the conception and design of the study, contributed to the analysis and interpretation of the data, and contributed to the drafts of this paper. All authors have read and approved the final manuscript.

## Role of funding source

This research was funded by 10.13039/501100001858Vinnova, Sweden’s Innovation Agency, Governmental Funding of Clinical Research within the National Health Service (ALF), 10.13039/501100009780Region Skåne and The Swedish Rheumatism Association, Malmö.

## Data references

The datasets used and/or analyzed during the current study are available from the corresponding author on reasonable request.

## Declaration of Competing Interest

The authors declare that they have no conflict of interests.
