# Supplementary material for: On the waiting list for joint replacement for knee osteoarthritis: Are first-line treatment recommendations implemented?
Source: Osteoarthr Cartil Open. 2020 Mar 10;2(2):100056. doi: 10.1016/j.ocarto.2020.100056 (PMC9718263; doi:10.1016/j.ocarto.2020.100056)
Supplement: Multimedia component [file mmc1.docx]

**Appendix A.** Questions asked translated from the Swedish version

Questions related to the treatment of your osteoarthritis before you were put on the waiting list for joint replacement

- **Have you been treated for your osteoarthritis before you were put on the waiting list?**

Yes ⎕ No⎕

- If **Yes,** what type of treatment:

BOA (Education and exercise with a physical therapist) ⎕

Physical therapy ⎕

Walking aid ⎕

Prescribed pain killers ⎕

Weight managment ⎕

Questions related to the recommendation of treatments of your osteoarthritis during your time on the waiting list for joint replacement

- **Have you been recommended other treatments for your osteoarthritis while waiting for your joint replacement?**

Yes ⎕ No⎕

- If **Yes,** what type of treatment were you recommended?

BOA (Education and exercise with a physical therapist) ⎕

Physical therapy ⎕

Walking aid ⎕

Prescribed pain killers ⎕

Weight managment ⎕

If you **never** have participated in BOA (Education and exercise with a physical therapist), would you be interested in participating in such management program while waiting for your joint replacement?

Yes ⎕ No⎕
